# Drug conjugates—an emerging approach to treat breast cancer

**DOI:** 10.1002/prp2.417

**Published:** 2018-07-05

**Authors:** Mahmud Hasan, Rehana K. Leak, Robert E. Stratford, Darius P. Zlotos, Paula A. Witt‐Enderby

**Affiliations:** ^1^ Division of Pharmaceutical, Administrative, and Social Sciences Duquesne University Pittsburgh PA USA; ^2^ Indiana University School of Medicine Indianapolis IN USA; ^3^ Department of Pharmaceutical Chemistry The German University in Cairo New Cairo City Cairo Egypt; ^4^ University of Pittsburgh Cancer Institute University of Pittsburgh Pittsburgh PA USA

**Keywords:** breast cancer, drug conjugates, hybrid ligands, nanoparticles

## Abstract

Breast cancer treatment using a single drug is associated with a high failure rate due, in part, to the heterogeneity of drug response within individuals, nonspecific target action, drug toxicity, and/or development of resistance. Use of dual‐drug therapies, including drug conjugates, may help overcome some of these roadblocks by more selective targeting of the cancer cell and by acting at multiple drug targets rather than one. Drug‐conjugate approaches include linking drugs to antibodies (antibody‐drug conjugates), radionuclides (radioimmunoconjugates), nanoparticles (nanoparticle‐drug conjugates), or to other drugs (drug‐drug conjugates). Although all of these conjugates might be designed as effective treatments against breast cancer, the focus of this review will be on drug‐drug conjugates because of the increase in versatility of these types of drugs with respect to mode of action at the level of the cancer cell either by creating a novel pharmacophore or by increasing the potency and/or efficacy of the drugs’ effects at their respective molecular targets. The development, synthesis, and pharmacological characteristics of drug‐drug conjugates will be discussed in the context of breast cancer with the hope of enhancing drug efficacy and reducing toxicities to improve patient quality of life.

## 1 INTRODUCTION

**Figure 1:**
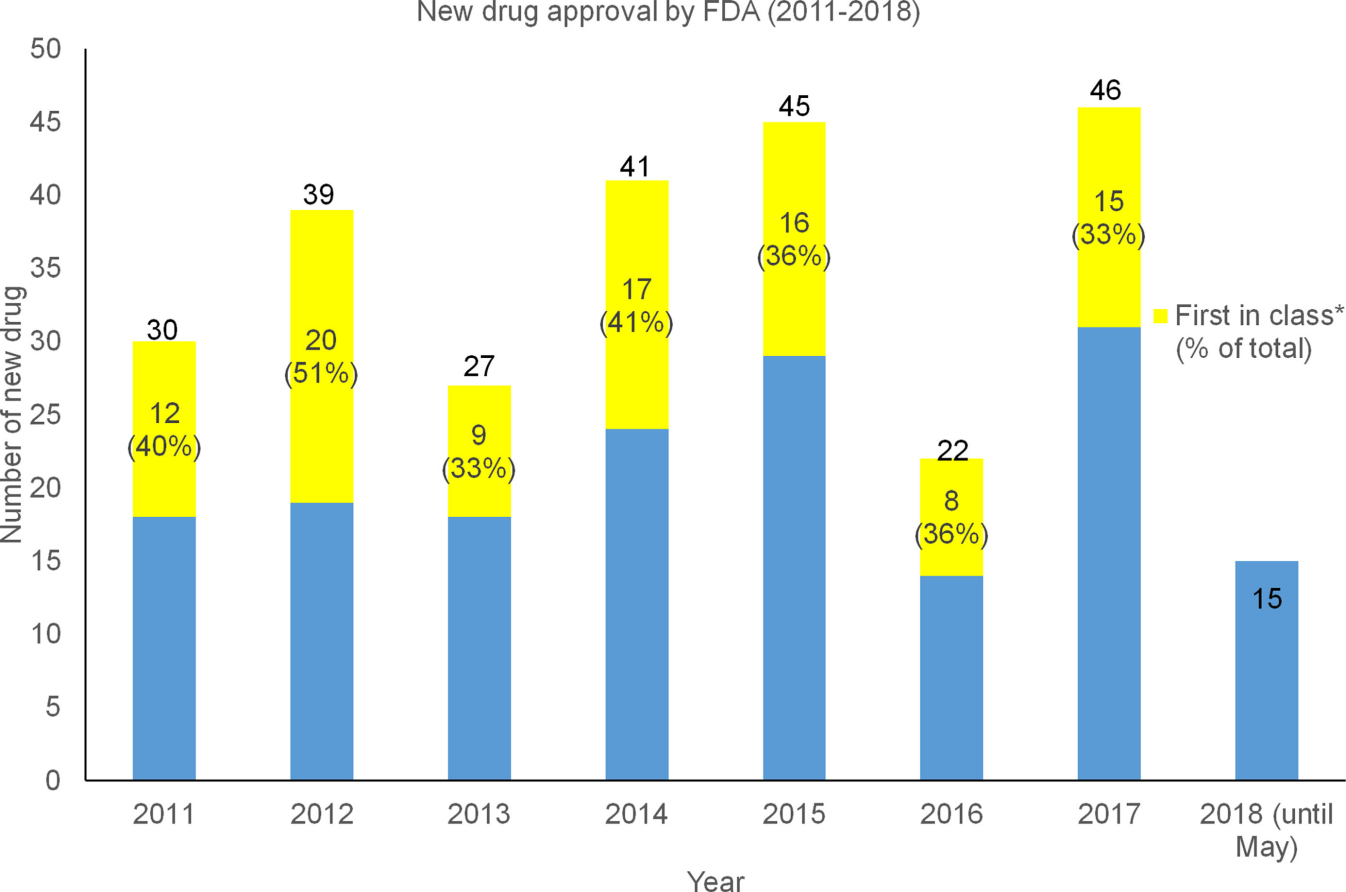
Number of new drugs approved by FDA from 2011 to 2018 (data collected from https://www.fda.gov/Drugs/DevelopmentApprovalProcess/DrugInnovation/default.htm)

Every year, the FDA approves a considerable number of new drugs, with 30%‐50% being first in a therapeutic class (Figure 1). The average cost for a new drug—from the synthesis to marketing—is approximately $2.5 billion, including drugs that meet with failure.^22^ During the clinical phases of drug development, failure often arises due to nonspecific target actions and poor bioavailability.^55^, ^60^ Many anticancer drugs target rapidly dividing cells, and drug toxicity may therefore occur in those tissues with high rates of proliferation (eg, bone marrow, oral cavity, skin, nails). More severe signs of toxicity include bone marrow depression and gastrointestinal, nervous system, hepatic, urinary tract, cardiac, or pulmonary toxicity. Metabolic abnormalities may also arise due to the lysis of dead cancer cells and the release of their intracellular contents.^91^ In addition to these safety challenges, there is no guarantee that a new preclinically validated drug will be efficacious when tested in the patient population.

Breast cancer is a complex disease and, unlike many other diseases, can readily develop drug resistance; this could be due to a set of gene mutations resulting in dysregulation of the balance between prosurvival and proapoptotic signaling cascades.^80^, ^105^ For example, patients with *BRCA1* and/or *BRCA2* gene mutations exhibit an increased risk of developing breast cancer.^36^ Mutation(s) of a specific signaling protein may also lead to drug resistance. For example, estrogen receptor‐positive breast cancer cells (MCF‐7) are known to display drug resistance by modulation of the antiapoptotic protein, Bcl2, in the presence of estrogenic stimulation.^108^ Resistance may also arise through activation of drug transporters that prevent the accumulation of the anticancer drug within the cell.^46^ For example, anthracycline drugs are readily effluxed from cancer cells overexpressing P‐glycoprotein or multidrug resistance protein.^27^ In addition, cancer phenotypes in different human populations vary, and a response to a single drug may not be equally efficacious across all individuals.^84^

**Figure 2:**
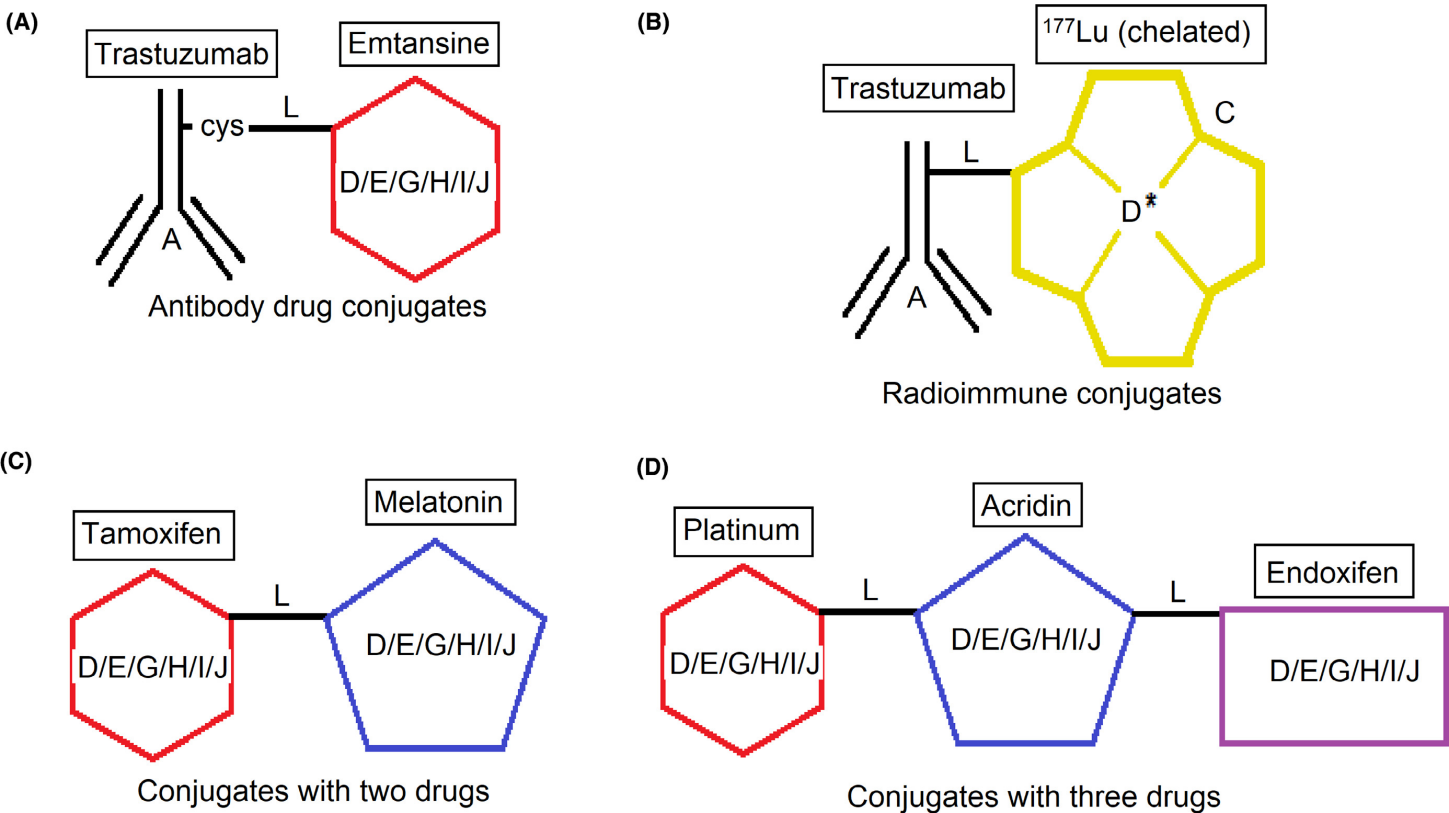
Possibilities of drug conjugates. A = antibody targets‐specific receptor (mentioned in table 1), B = radioactive isotope (mentioned in Table 2), C = chelating agent (DOTA, EDTA) D = cytotoxic drug (mentioned in Table 1), E = binds with a receptor (tamoxifen, endoxifen), F = regulates a signal pathway (anti‐NF‐КB, DNA intercalator), G = regulates an enzyme (kinase inhibitors, HDAC inhibitors), I = endogenous compound (melatonin), J = nanoparticle (gold). L = linker for conjugation (valine‐citrulline, hydrazine)

Drug conjugates represent a growing class of anticancer agents designed to (1) increase target selectivity and reduce the bystander effect (eg, drug‐antibody conjugates; Figure 2A; Table 1), (2) enhance cytotoxicity and tumor targeting (eg, radionuclide‐drug conjugates, nanoparticle‐drug conjugates; Figure 2B; Tables  2 and 3) or (3) enhance the potency and/or efficacy of anticancer therapy (eg, drug‐drug conjugates; Figure 2C; Table 4). To overcome low efficacy, drug resistance, and/or toxicity associated with single drug use or monotherapy, dual‐drug, and even triple‐drug therapies are being developed or are already in clinical use.^1^ For example, paclitaxel and trastuzumab combination drugs, or capecitabine and docetaxel combination drugs have been shown to reduce mortality and improve safety compared to their administration as single drugs.^78^ However, most of these therapies deliver drug combinations as separate entities.

**Table 1:** Antibody‐drug conjugates for breast cancer

Drug name	Status	Antibody, target	Cytotoxic drug, target	Target patients	Potency/Efficacy
Kadcyla^®^	Approved	Trastuzumab, HER2	Emtansine, antimicrotubule	HER2‐positive metastatic breast cancer	Improved overall survival compared to lapatinib plus capecitabine.^112^
Glembatumumab vedotin	Phase 2b	Glembatumumab, Glycoprotein NMB	Monomethyl auristatin E (MMAE), tubulin inhibitor	gpNMB overexpressing metastatic triple‐negative breast cancer	Well‐tolerated in pretreated patients.^122^
BMS‐182248‐1 (discontinued)	Phase 2	BR96, Lewis‐Y antigen	Doxorubicin, topoisomerase II inhibitor	Metastatic breast cancer	Limited clinical activity.^110^
IMMU‐132	Phase I	Sacituzumab govitecan, antitrop‐2	SN‐38, topoisomerase I inhibitor	Metastatic triple‐negative breast cancer	Well‐tolerated and robust response.^6^
BAY 1187982	Phase I	FGFR2, FGFR2 receptor	Auristatin, antimicrotubule	Cancer cells overexpressing FGFR	Effective compared to unconjugated antibodies in vitro^102^
SYD985	Phase I	Trastuzumab, HER2 receptor	Seco‐duocarmycin, DNA‐alkylating agent	BT‐474 cells and BT‐474 xenografted mice	Effective in BT‐474 xenografted in vivo model.^33^
Anti‐PTK7‐Aur0101	In vivo	Anti‐PTK7, antiprotein tyrosine kinase 7 antibody	Aur0101, microtubule inhibitor	Triple‐negative breast cancer	Induced sustained tumor regression.^17^
REGN2878‐DM1	In vivo	REGN2878, prolactin receptor	DM1, maytansine derivative	Prolactin receptor‐positive breast cancer	Significant antigen‐specific antitumor activity.^57^
DS‐8201a	In vivo	Trastuzumab, HER2 receptor	Exatecan derivatives, topoisomerase I inhibitor	Both HER2 + and HER2‐ breast cancer cells	Showed bystander toxicity.^83^
BT‐2111	In vivo	Trastuzumab, HER2 receptor	Melanotransferrin, cross blood‐brain barrier	Breast cancer metastasis to the brain in NuNu mice	68% reduction of metastasis in the brain compared to trastuzumab alone.^82^
FGF1_V_‐MMAE	In vitro	FGF1 receptor ligand variant (FGF1v), FGFR	MMAE, tubulin inhibitor	Cancer cells overexpressing FGFR	Conjugate showed higher potency than MMAE.^106^

Antibody‐cytotoxic drug conjugates (ADCs) are the most widely investigated drug conjugates to treat breast cancer.^89^ In general, a cytotoxic drug is attached to a monoclonal antibody that is specific for the target receptor (Figure 2A). The antibody binds to the receptor of the cancer cell where the cytotoxic drug is intended to exert its actions. Therefore, the cancer cells should ideally densely express the receptor for antibody binding. Although over 55 ADCs are currently in clinical trials,^13^ only 3 ADCs have been approved by the FDA.^77^ However, gemtuzumab ozogamicin (marketed as Mylotarg^®^ by Wyeth‐Ayerst) was withdrawn in 2010 due to increased patient mortality and demonstrating no clinical benefit over conventional therapy, which leaves only 2 ADCs available for clinical use. One of them is for HER2‐positive metastatic breast cancer—the trastuzumab‐emtansine conjugate marketed as Kadcyla^®^ by Genentech and Roche. Another one is brentuximab‐vedotin (Adcetris^®^ marketed by Seattle Genetics) for Hodgkin lymphoma or anaplastic large cell lymphoma. In many ways, ADCs may exert potential benefits over conventional treatment. For example, highly cytotoxic drugs might become safer for normal cells when they are bound to cancer cell‐specific antibodies.^115^

**Table 2:** Potential radionuclide conjugates for breast cancer

Isotope	Status	Antibody	Model	Potency/Efficacy
^177^Lu (chelated)	Pilot clinical study	Trastuzumab	HER2‐positive breast cancer patients	No drug uptake for HER2‐negative patients, whereas the radioimmunoconjugate retains its integrity up to 7 days in vivo.^8^
^213^Bi (chelated)	In vivo	Antibody to human aspartyl β‐hydroxylase	4T1 tumor mice	Significant effect on the tumor.^92^
^213^Bi (chelated)	In vivo	Antibody to chondroitin sulfate proteoglycan 4	TNBC xenograft and in vitro	Significantly inhibited tumor and cell growth.^53^
^225^Ac (chelated)	Anti‐rat HER2′/In vivo	Anti‐rat HER2	Metastatic breast cancer mouse model	Complete eradication of lung metastasis and more efficacious than^213^Bi.^103^
^111^In/^90^Y (peptide linked)	Phase I	M170	Advanced breast cancer patients	Patients had grade 3 or 4 myelosuppression^93^
^213^Bi (chelated)	In vivo	Plasminogen activator inhibitor‐2	MDA‐MB‐231 cell inoculation in mice	Inhibit growth at 2 days after inoculation.^2^
^90^Y (chelated)	In vivo	Cilengitide	HBT 3477 cell xenografted mice	Increased efficacy compared to radiotherapy/cilengitide alone^9^
^131^I	In vivo	Humanized anti‐Lewis Y	MCF‐7 xenografted mouse	Significant tumor growth inhibition compared to control radiolabeled antibody^15^
^131^I	In vivo	^131^I‐IgG2a (rat)	MDA‐MB‐361 xenograft	Tumor growth inhibition for more than 24 days 101
^111^In/^90^Y	Phase I	IgG1 (BrE3)	Metastatic breast cancer patients	Risk of developing HAMA response 19
^131^I	Pilot clinical study	^131^I‐IgG1 (ChL6)	Metastatic breast cancer patients	Partial response achieved with the development of thrombocytopenia and granulocytopenia^20^, ^21^

Radiation emitted from a radionuclide can be used to kill cells. Radioactive compounds can also attack noncancerous cells; therefore, targeted delivery of the radionuclide with the help of a monoclonal antibody is desirable. The radionuclide can be linked to a monoclonal antibody by a linker, or the antibody could be labeled with radioisotope by a chelation method (Figure 2B).

**Table 3:** Drug‐delivery system conjugates

Drug	Status	Delivery system	Model	Efficacy/Potency
Tamoxifen	In vivo	Naringen (P‐gp efflux inhibitor)	MCF‐7 cells and female Wistar rats	The conjugate showed 22‐fold increased cytotoxicity compared to tamoxifen or the combination.^95^
Tamoxifen	In vivo	Chitosan‐stearic acid‐based polymeric micelles	MCF‐7 cells	Enhanced cytotoxicity and modified pharmacokinetic profiles.^109^
Tamoxifen	In vitro	Trans‐2‐phenylcyclopropylamine	Lysine‐specific demethylase 1‐triggered controlled release	No toxic effect on normal cells.^85^
Tamoxifen	In vitro	Glucosamine‐porphyrin	MCF‐7 cells	Works through necrosis/apoptosis pathways.^4^
Tamoxifen	In vivo	Bile (cholic) acid	4T1 in vivo model	More potent than tamoxifen.^104^
Tamoxifen	In vitro	Thiol‐polyethylene glycol gold nanoparticle	MCF‐7 cells	The conjugate showed 2.7 folds higher potency than tamoxifen with less cytotoxicity to cancer cells.^28^
Tamoxifen	In vitro	Pyropheophorbide	MCF‐7 cells	Showed light‐specific cytotoxicity^35^
Gefitinib	In vitro	Polyarginine peptoids	MDA‐MB‐468, NME, and LM1 cell lines	NArg‐based conjugate blocked STAT3 phosphorylation without affecting ERK1/2^7^
Mitoxantrone	In vivo	Folic acid‐tocopheryl polyethylene glycol	MCF‐7 xenografted mice	MTO‐FMCT showed improved cellular uptake with higher MCF‐7 cytotoxicity. MTO‐FMCT showed higher potency to reduce MCF‐7 cell viability compared to MTO alone^43^
Polymeric doxorubicin	In vivo	Aminopropyltriethoxysilane‐modified porous silicon particles	MDA‐MB‐231 and 4T1 mouse models of metastatic breast cancer	Nanoparticles showed enhanced efficacy with functional cures in 40%‐50% of treated mice^120^
Doxorubicin	Retrospective Clinical Study	Pegylated liposomal nanoparticles	Stage I‐III triple‐negative breast cancer patients	Adjuvant chemotherapy was as effective as conventional chemotherapy with reduced toxicity^66^
Paclitaxel (Abraxane^®^)	FDA approved	Albumin‐bound nanoparticles	Clinical trials on metastatic breast cancer	Abraxane^®^ showed superior efficacy and reduced toxicity compared with paclitaxel^39^

The uptake of nanoparticles by a tissue depends on the hydrophobicity of that nanoparticle. For example, nanoparticles deposited in certain organs such as the liver, spleen, and reticuloendothelial system correlate positively with the increasing hydrophobicity of the polymer.^37^ Although several nanoparticle‐based drug delivery systems have been developed, only albumin‐bound paclitaxel nanoparticle (Abraxane^®^) was approved by FDA for metastatic breast cancer and nonsmall cell lung carcinoma.^69^ Nanotechnology can also be effectively used in breast cancer treatment. Nanoparticle conjugates may show increased potency by penetrating the cells by endocytosis instead of the diffusion method used for a single drug.^28^ This could be a mechanism to avoid efflux by drug transporters such as P‐glycoprotein.^14^ In a phase III clinical trial, paclitaxel nanoparticles bonded with albumin showed superior efficacy and safety compared to paclitaxel dissolved in castor oil.^39^ Furthermore, doxorubicin linked with poly(L‐glutamic acid) by a pH‐sensitive cleavable linker showed enhanced efficacy in MDA‐MB‐231 and 4T1 metastatic breast cancer mouse model.^120^

**Table 4:** Hybrid drug conjugates targeting breast cancer

**Drug conjugate/Drug class**	**Status/model**	**Potency/efficacy**
Ribociclib‐vorinostat/cyclic‐dependent kinase CDK‐4–HDAC inhibitor 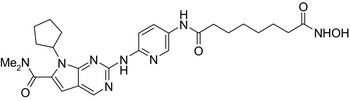	In vitro MDA‐MB‐231 cellsIn vivo 4T1 cells of rat breast cancer	Conjugate showed higher cytotoxicity on MDA‐MB‐231 cells (IC_50_ = 1.86 μmol/L) than vorinostat (IC_50_ = 2.59 μmol/L) and ribociclib (IC_50_ > 10 μmol/L) and stronger tumor growth inhibition in 4T1 cells (79%) than vorinostat (75.6%) and ribociclib (38.9%)^65^
Fibroblast growth factor 1 inhibitor‐nexturastat/FGFR 1‐HDAC‐6 inhibitor 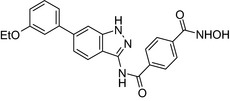	In vitro MCF‐7 cells	Conjugate showed cytotoxic activity on MCF‐7 cells (IC_50_ = 9 μmol/L)^67^
Raloxifen‐dimethyl fumarate/SERM–anti‐NF‐κB 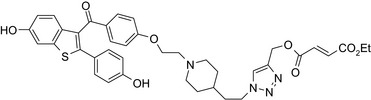	In vitro MCF‐7 cells	Higher inhibition of NF‐κB than fumarate alone^54^
Olaparib‐vorinostat/PARP inhibitor–HDAC inhibitor 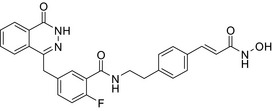	In vitro MDA‐MB‐231 and HCC1937 cells	Conjugate showed more potent activity than olaparib and vorinostat with 4.1‐fold less cytotoxicity to MCF‐10A^123^
Ruxolitinib‐vorinostat/Janus kinase‐HDAC inhibitor 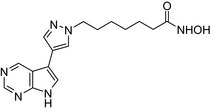	In vitro MCF‐7 cells	Conjugate was equipotent on MCF‐7 cells (IC_50_ = 0.84 μmol/L) to vorinosta (IC_50_ = 0.84 μmol/L) and more potent than ruxolitinib (IC_50_ = 10 μmol/L)^121^
Combretastatin‐cyclofenil/Antimitotic‐SERM 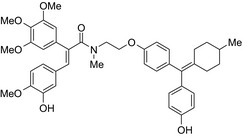	In vitro MCF‐7 cells	Cyclofenil‐combretastatin conjugate (IC_50_ = 187 nmol/L) showed potent antiproliferative activity to MCF‐7 cells^56^
Combretastatin endoxifen/Antimitotic‐SERM 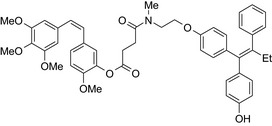	In vitro MCF‐7 cells	Endoxifen‐combretastatin conjugate (IC_50_ = 5.7 nmol/L) showed potent antiproliferative activity to MCF‐7 cells^56^
Endoxifen‐combretastatin/Antimitotic‐SERM 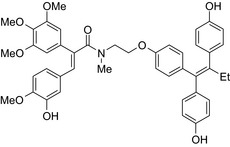	In vitro MCF‐7 and MDA‐MB‐231 cells	The conjugate showed potent antiproliferative activity (IC_50_ = 5 nmol/L) to MCF‐7 cells^56^
Vandetanib‐vorinostat/VEGFR‐HDAC inhibitor 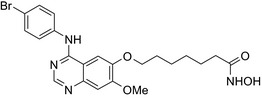	In vitro MCF‐7 cells	Conjugate was more potent on MCF‐7 cells (IC_50_ = 0.85 μmol/L) than vandetanib (IC_50_ = 18.5 μmol/L) and vorinostat (IC_50_ = 4.5 μmol/L)^87^
TBB‐triazole hydroxamic acid/Casein kinase 2–HDAC inhibitor 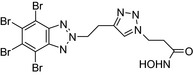	In vitro MCF‐7 cells	The conjugate showed cytotoxic activity (IC_50_ = 4.26 μmol/L) on MCF‐7 cells^90^
Oxabicycloheptene sulfonate‐vorinostat/ERα antagonist–HDAC inhibitor 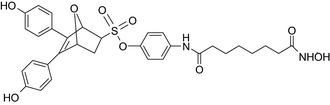	In vitro MCF‐7 cells	The conjugate showed higher potency than tamoxifen.^107^
ICI‐164,384‐N‐butylvorinostat/ER antagonist‐HDAC inhibitor 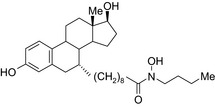	In vitro MCF‐7 and MDA‐MB‐237 cells	Conjugate was more potent on MCF‐7 cells (IC_50_ = 0.34 μmol/L) than ICI‐164,384 (IC_50_ = 0.93 μmol/L) and vorinostat (IC_50_ = 0.32 μmol/L)^76^
Semaxanib‐vorinostat / VEGFR‐HDAC inhibitor 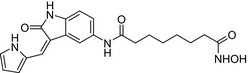	In vitro MDA‐MB‐237 cells	Conjugate was equipotent on MDA‐MB‐237 cells (IC_50_ = 117 nmol/L) to vorinostat (IC_50_ = 118 nmol/L)^86^
Melatonin‐tamoxifen/SERM–melatonin receptor agonist 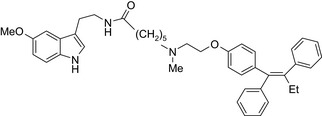	In vitro BC cells In vivo ovariectomized FVB/n mice	Hybrid conjugate did not increase uterus weight compared to tamoxifen, and showed efficacy against different BC cells including tamoxifen‐resistant MCF‐7 cells, to be published,^116^ US patent no. ‐ 08785501)
Colchicin‐pironetin/Β‐tubulin inhibitor–α‐tubulin inhibitor 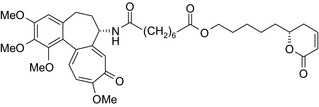	In vitro MCF‐7 cells	All conjugates showed lower cytotoxicity values than the parental molecules, whereas the binding of the conjugates to tubulin depends on the length of the linkers^113^
c‐Src kinase inhibitor vorinostat/c‐Src‐HDAC inhibitor 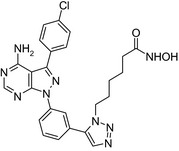	In vitro SK‐BR‐3 cells	Conjugate was more potent on SK‐BR‐3 (IC_50_ = 0.2 μmol/L) than vorinostat (IC_50_ = 1.2 μmol/L)^59^
Platinum‐acridin‐endoxifen/DNA intercalation & platination–SERM 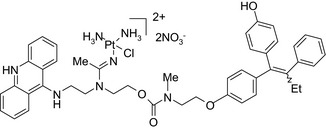	In vitro MCF‐7 and MDA‐MB‐231 cells	One conjugate showed higher potency on MCF‐7 cells compared to cisplatin or tamoxifen^23^
Endoxifen‐endoxifen/Bivalent SERM 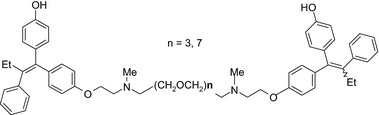	In vitro MCF‐7 and MDA‐MB‐231 cells	Bivalent ligands showed higher potency than 4OH tamoxifen^98^
Tamoxifen‐vorinostat/SERM‐HDAC inhibitor 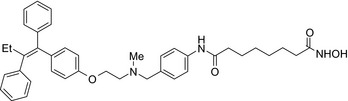	In vitroMCF‐7 and MDA‐MB‐231 cells	The conjugate showed higher cytotoxicity on MCF‐7 (IC_50_ = 3.8 μmol/L) and on MDA‐MB‐231 cells (IC_50_ = 8.1 μmol/L) than tamoxifen and vorinostat^40^
Doxorubicin‐RU 39411/Topoisomerase inhibitor–ER antagonist 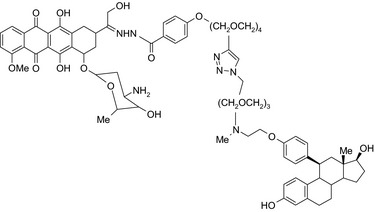	In vitro MCF‐7 and MDA‐MB‐231 cells	The conjugate was about 70‐fold more potent than doxorubicin to inhibit MCF‐7 cell proliferation^18^
Erlotinib‐vorinostat CUDC‐101/EGFR‐HER2‐HDAC inhibitor 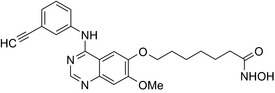	In vitro MCF‐7 and MDA‐MB‐231 cellsIn vivo xenograft mice	Conjugate was more potent on MCF‐7 cells (IC_50_ = 0.55 μmol/L) than erlotinib (IC_50_ = 20 μmol/L) and vorinostat (IC_50_ = 2.8 μmol/L) and the combination of the parent drugs (IC_50_ = 2.7 μmol/L)^64^
Lapitanib‐panobinostat/EGFR‐HER2‐HDAC inhibitor 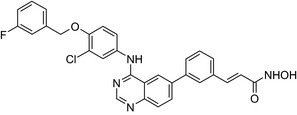	In vitroSKBR3 cells	Conjugate is more potent on SMBR3 cells than lapitanib and vorinostat^70^
Estradiol‐cisplatin/ER agonist–antineoplastic 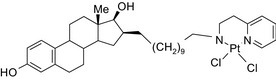	In vivoMCF‐7 and MDA‐MB‐468 mouse xenografts	The conjugates decreased tumor volume compared to cisplatin in ER‐positive mice^111^
Retinoic acid‐butyric acid/RAR & RXR agonist–HDAC inhibitor 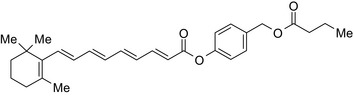	In vitroMCF‐7 andMDA‐MB‐231 cells	The conjugate showed 1085‐fold higher potency than parent retinoic acid and 100000‐fold higher potency than butyric acid^38^
Tamoxifen‐ferrocene/SERM–organometallic complex 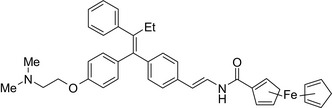	In vitroMCF‐7 cells	Increased apoptotic events compared to tamoxifen/ferrocene^117^
Doxorubicin‐4OH tamoxifen/Topoisomerase inhibitor–SERM 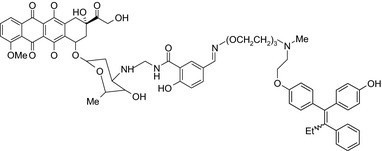	In vitroMCF‐7, MCF‐7 resistant,MDA‐MB‐231, MDA‐MB‐435 cells	The conjugates showed 4‐ to 140‐fold higher potency than doxorubicin^10^
Aniline mustard‐estradiol/DNA‐alkylating agent–ER agonist 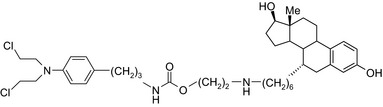	In vitroMCF‐7 and MDA‐MB‐231 cells	The conjugate showed higherpotency compared to chlorambucil^79^
Aniline mustard‐phenylindole/DNA‐alkylating agent–SERM 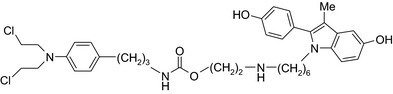	In vitroMCF‐7 and MDA‐MB‐231 cells	Two conjugates showed higher toxicity to MCF‐7 than to MDA‐MB‐231 cells^94^

SERM, Selective estrogen receptor modulator, GPCR, G protein‐coupled receptor.

Drug conjugates offer an alternative approach in situations where a single drug or monotherapy fails. A drug‐drug conjugate comprises 2 or more drugs connected by a chemical linker (Figure 2C and D). These novel types of drugs may be useful for targeted drug delivery and modulation of pharmacokinetic parameters, rendering the drugs more effective and less toxic than in isolation. Furthermore, conjugation of 2 drugs (eg, melatonin‐tamoxifen) with a noncleavable linker may produce a novel molecule with unique pharmacological characteristics,^116^ (US Patent No. 08785501). Although antibody‐drug conjugates comprise a large segment of conjugated drugs (see Table 1), this class of drugs will not be discussed here, and the reader is referred to recent excellent papers.^51^, ^89^ The focus of this review will be on the emerging field of drug‐drug conjugates reported in the literature, their design and their pharmacological characteristics with respect to drug targets (receptors, intracellular signaling proteins) and efficacy as antibreast cancer agents.

## 2 DRUG CONJUGATES/HYBRID LIGANDS

Ideally, a drug‐drug conjugate should be stable in the systemic circulation before arriving at the target site, which is not only valid for drug conjugates with a noncleavable linker, but also for those formed by a cleavable spacer such that drug release will occur only at the intended site of action.^26^ A noncleavable linker may also be designed to maintain its chemical integrity, allowing it to act as a single moiety/pharmacophore at its receptor or receptors.^72^ Unlike combination drug therapy, in which 2 individual unlinked drugs are administered simultaneously, conjugated drug‐drug therapies may offer distinct advantages therapeutically, aesthetically, or both.^63^ With respect to esthetics, it may be more convenient for a patient to take a single drug conjugate instead of 2 drugs, thus improving compliance.^63^ Concerning therapeutic advantages, conjugated drug therapies may improve the therapeutic index by lowering the minimum effective dose and by increasing the maximum tolerated dose compared to traditional combination therapies.^73^ Drug‐drug conjugates may also prevent drug resistance, as tumors are less likely to become resistant to drugs with distinct targets (ie, receptors, and/or intracellular signaling proteins).^1^, ^16^ A drug conjugate may display superior activity (potency, and/or efficacy) compared to a single drug; this may be a result of improved bioavailability^119^ or through additive or synergistic action of the 2 drugs working in concert.^97^ Moreover, 2 conjugated pharmacophores targeted for 2 different receptors may show higher affinity by coactivation in the same cells.^48^ For example, paclitaxel and doxorubicin drug conjugates showed superior efficacy and safety compared to the combination by acting synergistically because it delivers both drugs to the same target site at a synergistic ratio.^71^ The increase in potency of a drug‐drug conjugate may also produce less toxicity as the recommended dose could be lowered.^5^, ^24^, ^61^, ^62^ For example, chimeric c‐Src kinase and histone deacetylase inhibitors^59^ and the multiacting EGFR/HER2 and histone deacetylase inhibitor CUDC‐101^12^ were reported to be not only more active than the combination of the 2 constituting parent compounds, but also less toxic with a higher improved therapeutic index. These findings indicate that molecular hybridization does not necessarily lead to the additive toxicity often observed for the combination therapy.

## 3 DESIGNING DRUG CONJUGATES

### 3.1 Drug targets

Stability and efficacy of drug conjugates need to be carefully considered during their design. Concerning stability, conjugates containing a cleavable linker should be designed to release drugs only at the target site.^24^ With respect to improved efficacy, conjugates can be designed to target breast tumors with unique phenotypes, such as luminal A (ER+/HER2+; PR+/HER2−), luminal B (ER+/HER2+; PR+/HER2+), HER2 (ER‐/PR‐/HER2+), or to target specific intracellular signaling proteins in triple‐negative breast cancer, (eg, mTOR, PI3K, PARP, TROP‐2, VEGFR, EGFR, FGFR, CYP17A1, MEK, AKT, anti‐CD27, anti‐CD52, hedgehog).^124^ In such cases, combining 2 drugs with different pharmacological targets ensures their equivalent delivery to the cancer tissue, which may not be the case if the 2 combination drugs differ in pharmacokinetic properties of tissue distribution and elimination.

### 3.2 Linker design

The following questions need to be addressed when designing chemical linkers: Should it be stable in the circulation? Should it release the 2 active drugs at the target site? Should it be stable at the target site? Should it influence drug activity? Stable linkers can be simple (CH_2_)_n_‐chains (eg, tamoxifen‐melatonin drug conjugate or more hydrophilic amides (eg, valine‐citrulline linker), and ether‐containing spacers (eg, maleimidomethyl cyclohexane‐1‐carboxylate linker); the design depends on the drugs themselves and sites of attachment; and on the desired action on the tumor.^24^, ^74^, ^116^ Furthermore, if the efficacy of the drug conjugate is dependent upon linker stability, then noncleavable linkers may be superior because they can only be degraded once they are internalized by lysosomes within cells.^74^ However, it is also possible that the conjugate will exert its action by maintaining the integrity of the linker until it reaches its target site(s) where it will then be broken down chemically or enzymatically depending on the structure of the linker; this type of linker is referred to as a cleavable linker.^74^ One drawback to the type of drug conjugate is that the local release of drugs at their target site(s) may also impact neighboring “nontarget” sites, which may cause toxicity depending upon the cellular milieu.^47^

Drugs conjugated with a chemically cleavable linker are released under specific biochemical environments. A hydrazone linker is usually stable in standard blood pH conditions (pH 7.4) and hydrolyzed under acidic conditions, such as in lysosomes or in the more acidic tumor microenvironment.^30^ For example, adriamycin conjugated to PEO‐b‐PAsp copolymers with a hydrazone linker exhibited release of the active drug at pH levels below 5.^44^ However, one drawback to this type of linker may be its instability in acidic buffers and excipients as observed for hydrazone linkers.^52^ In a recent study, a drug‐antibody conjugate using this type of linker [ie, gemtuzumab ozogamicin (Mylotarg^®^)] was withdrawn due to its narrow therapeutic index, poor plasma stability, and lack of benefit over conventional chemotherapy.^30^, ^74^, ^88^ Even though this was with a drug‐antibody conjugate, these findings need to be taken into consideration when designing a drug‐drug conjugate.

Chemical linkers containing disulfide bonds are also chemically cleavable. For example, disulfide bonds may be reduced in vivo by glutathione. As glutathione levels are relatively low in plasma (2‐20 μmol/L) compared to the cytoplasm (0.5‐10 mmol/L)^75^, ^115^, ^118^ disulfide bond‐containing linkers remain relatively stable in blood. This property improves the pharmacokinetic profiles of the conjugates compared to their unconjugated antibody component. For example, the disulfide linker containing a doxorubicin‐gold drug conjugate exhibited greater intracellular uptake than free doxorubicin in multidrug‐resistant cancer cells.^41^ One point to consider when creating drug conjugates with a disulfide linker is its stability during the sterilization process. Heat generated during the sterilization process can break down disulfide bonds.^68^ This obstacle could be overcome through filter sterilization.^31^

Linkers may be designed to be degraded by enzymes within the breast cancer cell to control drug delivery at the target site; this may avoid premature release into the systemic circulation due to factors such as low pH.^30^ For example, lysosomal enzymes located in breast cancer cells can be leveraged to design conjugates that will only release the drug intracellularly. A commonly used peptide linker, valine‐citrulline, is cleaved by cathepsin B and exhibits superior stability compared to the hydrazone linker.^25^ For example, a drug‐antibody conjugate (ie, a Lewis Y‐specific antibody conjugated to monomethyl auristatin E) connected by a valine‐citrulline linker showed superior stability in buffer and plasma, suggesting that the conjugate will release the drug only in the presence of cathepsin.^25^ In another study, it was shown that the valine‐citrulline linker exhibits greater stability compared to the Phe‐Lys peptide linker in the presence of cathepsin B alone, as doxorubicin release from the conjugate was 30‐fold faster for the Phe‐Lys linker than the valine‐citrulline linker.^29^ Glucuronide‐based linkers can also be used as nonpeptide linkers that release drugs within the interior of the cell through the action of lysosomal β‐glucuronidases.^11^, ^50^, ^74^ For example, a camptothecin analog conjugated to a CD30 or Lewis Y antibody with a glucuronide linker demonstrated greater potency to inhibit breast cancer cell viability compared to these same drugs connected using a dipeptide linker.^11^ Even though these examples were based on drug‐antibody conjugates, these approaches may also be useful in designing drug‐drug conjugates.

Attention should also be drawn to the attachment points of the linker to the parent drugs. In particular, the chemical nature and attachment positions of the functional groups connecting the 2 pharmacophores should be chosen in accordance with the structure‐activity relationships with the given target to maintain the pharmacological actions of the 2 constituents in the dually acting molecular hybrid.^81^

## 4 PHARMACOLOGY OF DRUG‐DRUG CONJUGATES

Drug conjugates (molecular hybrids, chimeric drugs) are compounds that incorporate 2 drugs into a single molecule (Figure 2C). The 2 pharmacophores could be either directly linked or connected by a long cleavable/noncleavable linker/spacer described previously. Drug conjugates are expected to exert simultaneous action at the biological targets specific to each drug, which may enhance their potency, and/or efficacy compared to their administration as 2 individual agents. The efficacy can be improved due to either an increased potency of the conjugate or by improving pharmacokinetic parameters. For example, ribociclib‐vorinostat drug conjugates demonstrated higher potency (IC_50_ = 1.86 μmol/L) than vorinostat (IC_50_ = 2.59 μmol/L) and ribociclib (IC_50_ > 10 μmol/L) in the triple‐negative breast cancer cells, MDA‐MB‐231.^65^ Although this class of conjugates is not yet available on the market, some show promise as breast cancer drugs both in vivo and in vitro*,* as described in Table 4.

Antiestrogen/cytotoxic drug conjugates are commonly researched against ER+ type breast cancer, in which estrogen plays an indispensable role in tumor progression. Tamoxifen is the most commonly used antiestrogen drug for the synthesis of the anticancer drug hybrids (Tables 3 and 4). For example, a tamoxifen‐melatonin drug conjugate with similar affinity to estrogen receptors and MT1 melatonin receptors as tamoxifen or melatonin, respectively, demonstrated unique pharmacological characteristics at higher (>10 μmol/L) concentrations where the total number of estrogen receptor and MT1 melatonin receptor ligand‐binding sites increased compared to the decreases observed in the presence of tamoxifen or melatonin alone. Because this was not observed for tamoxifen alone or melatonin alone, this suggests that the melatonin‐tamoxifen conjugate may be creating a unique pharmacophore between estrogen receptors and MT1 melatonin receptors. This is underscored in an in vivo study in female mice where tamoxifen alone and tamoxifen plus melatonin (coadministered but unlinked) increased uterine weight while the melatonin‐tamoxifen conjugate was without effect and similar to control mice,^116^ (US Patent No. 08785501). The melatonin‐tamoxifen conjugates may be creating a unique pharmacophore in mouse uterus preventing it from excessive stimulation by tamoxifen.

Histone deacetylase (HDAC) inhibitors were reported to show promising activity in triple‐negative breast cancer.^34^ Indeed, linking of tamoxifen to the histone deacetylase inhibitor vorinostat resulted in a conjugate with higher cytotoxicity in MCF‐7 and MDA‐MB‐231 cells than the parent drugs.^40^ The RU39411‐like antiestrogen compound conjugated with doxorubicin exhibited lower IC_50_ values (ie, greater potency) compared to doxorubicin only or doxorubicin plus the linker when tested in MCF‐7 cells, but not MDA‐MB‐231 cells.^18^ The triple drug conjugate platinum‐acridine‐endoxifen exhibited higher cytotoxicity compared to a tamoxifen/cisplatin combination therapy, which warrants further investigation.^23^ Another triple conjugate, 4‐hydroxytamoxifen‐doxorubicin‐formaldehyde showed increased drug uptake in estrogen receptor‐positive breast cancer cell lines compared to untargeted doxorubicin−formaldehyde conjugates.^10^ These studies further support the versatility and therapeutic efficacy of drug conjugates, which can be expanded from a dual‐drug approach to a triple‐drug conjugate approach.

Bivalent ligands (2 identical pharmacophores connected by a spacer) can also serve as drug conjugates. For example, bivalent ligands of hydroxytamoxifen—an estrogen receptor antagonist in breast tissue—exhibited more potent (IC_50_ ≤ 0.11 μmol/L) growth inhibitory effects in MCF‐7 cells compared to monomeric hydroxytamoxifen (IC_50_ = 0.15 μmol/L); in the same study, bivalent ligation with an estrogen receptor agonist (diethylstilbestrol) did not show any effect.^98^ The activity of the bivalent conjugate depends on the length of the spacer with a maximal estrogen receptor binding achieved for the (CH_2_OCH_2_)*n*‐linked endoxifen‐endoxifen bivalent ligands with an n of 3 or 7^98^; Table 4).

An estradiol‐cisplatin hybrid conjugate showed antiproliferative activity in the low micromolar range, which was more potent than cisplatin alone in estrogen‐dependent and estrogen‐independent breast cancer cell lines MCF‐7 and MDA‐MB‐231, respectively.^111^ Additionally, this conjugate displayed high affinity toward the estrogen receptor α and was superior to cisplatin in inducing tumor regression in the MCF‐7 human breast cancer tumors in a mouse model. Recently, conjugates of selective estrogen receptor modulators (ie, endoxifen or cyclofenil) linked to combretastatin, an antimitotic agent that binds to the beta subunit of tubulin, demonstrated potent (nmol/L) antiproliferative activity against MCF‐7 cells (Table 4).^56^, ^58^

An estrogen receptor antagonist, oxabicycloheptene sulfonate, linked to the histone deacetylase (HDAC) inhibitor vorinostat (suberanilohydroxamic acid) showed higher antitumor potency in MCF‐7 cells compared to the oxabicycloheptene sulfonate alone, indicating that parallel inhibition of HDAC and the estrogen receptor is beneficial for anticancer action.^107^

DNA‐alkylating agents linked to estrogen receptor ligands is yet another approach to developing novel hybrid drugs targeting specific phenotypes of breast cancer.^94^ For example, a hybrid drug conjugate consisting of an aniline nitrogen mustard with an estrogen receptor ligand belonging to the class of 2‐phenylindoles was reported to display selective toxicity toward estrogen receptor‐positive MCF‐7 cells compared with the estrogen receptor‐negative line, MDA‐MB‐231.^94^ Drug conjugates created by replacing the phenylindole group with estradiol and combining this to bis‐chloroethyl aniline mustard demonstrated selective toxicity toward estrogen receptor‐positive cancer cells.^79^, ^99^

The HDAC inhibitors, butyric acid and MS‐275 (entinostat), were each conjugated to *trans*‐retinoic acid via an ester linkage.^3^ Trans‐retinoic acid induces differentiation and arrests proliferation in a wide range of cancer cells.^96^ The 2 retinoic acid conjugates displayed potent antiproliferative activity in a hormone‐insensitive breast cancer cell line, MDA‐MB‐231 and some drug‐resistant breast cancer cell lines, MCF‐7_TAMR_, MCF‐7_ HOX‐B7_, LTLC and LTLT‐Ca.^38^

Another drug conjugate linked a β‐tubulin inhibitor, colchicine, to an analog of the α‐tubulin‐binding agent, pironetin, through an ester‐amide spacer. Although colchicine and the pironetin analog administered individually displayed similar toxicity toward the breast adenocarcinoma (MCF‐7) and the nontumoral HEK‐293 cell lines, the colchicine‐pironetin drug conjugate was 100‐fold more toxic to the MCF‐7 cell lines than either parent drug^113^ perhaps due to enhanced antimitotic actions in the more rapidly dividing cancer cell.

A conjugate consisting of vorinostat (an HDAC type I and II inhibitor) with olaparib [poly (ADP‐ribose) polymerase (PARP) inhibitor] demonstrated higher antiproliferative activity than olaparib or vorinostat alone in a variety of cancer cells, including breast cancer (MDA‐MB‐231, HCC1937) and Burkitt's lymphoma cells. This vorinostat‐olaparib drug conjugate also demonstrated lower cytotoxicity in normal mammary (MCF‐10A) cells compared to vorinostat, indicating that simultaneous targeting of PARP and HDAC might be beneficial for breast cancer therapy with a better safety profile.^123^

Many molecular hybrids have been readily designed for cytotoxic activity against a variety of cancer cells, such as the dual protein tyrosine kinase—HDAC inhibitors. For a more detailed report on chimeric HDAC inhibitors, the reader is referred to a very recent comprehensive review.^45^ The clinically most advanced agent is a multitarget EGFR/HER2‐HDAC inhibitor CUDC‐101,^12^, ^114^) that reached phase‐1 clinical trial in patients with advanced solid tumors.^100^ CUDC‐101 was more potent (IC_50_ = 0.55 μmol/L) than the combination of the parent drugs erlotinib + vorinostat (2.7 μmol/L) in MCF‐7 cells and promoted tumor inhibition in various cancer xenograph models including breast cancer.^64^ Other dual protein tyrosine kinase—HDAC inhibitors (Table 4) that were reported to display higher or equal cytotoxicity compared to the parent agents in various breast cancer cell lines include hybrids of ribociclib (CDK‐4 inhibitor) and vorinostat, FGFR‐1‐inhibitor and nexturastat,^67^ ruxolitinib (Janus kinase inhibitor)‐vorinostat,^121^ vandetanib (VEGFR inhibitor)–vorinostat,^87^ casein kinase 2 inhibitor and triazole hydroxamic acid (HDAC inhibitor),^90^ semaxanib (VEGFR inhibitor) and vorinostat,^86^ c‐Src kinase inhibitor and vorinostat,^59^ and lapitanib (EGFR inhibitor) and panabinostat.^70^

The concept of drug conjugates can also be used in the development of targeted drug delivery systems. Targeted drug delivery systems are generally stable in the systemic circulation, and they might be designed to release the cytotoxic drugs only after internalization into cancer cells.^49^ Some drug conjugates were specifically designed to deliver the cytotoxic drug at the target site using a pharmacologically inactive drug. Examples of tamoxifen drug conjugates targeted for specific drug delivery are described in Table 3.

## 5 CONCLUSION

Hundreds of receptors and signaling pathways are dysregulated in breast cancer, a highly heterogeneous disease that exploits multiple targets, such as receptors from different classes and myriad signal transduction cascades.^42^ Because of these factors, breast cancer drugs need to be selective and personalized based on the patient's genotype or phenotype. Drug‐drug conjugates offer a partial solution to these issues because conceptually they target multiple proteins and signaling pathways at the same time within a cancer cell. For 2 drugs with very different pharmacokinetic properties of distribution and elimination, it may not be practical to achieve therapeutic concentrations in a tumor at the same time, unless the drugs are chemically linked. Although only 32 unique generic medications are currently approved to prevent and treat breast cancer (data collected from https://www.cancer.gov/about-cancer/treatment/drugs/breast), there are 496 drug conjugate possibilities if 2 of the 32 drugs are linked. As summarized in Tables 1, 2, 3, 4, several in vitro and preclinical examples suggest that conjugates may harbor a therapeutic advantage over a single drug or standard drug combination treatment regimens.

As almost 60% of breast cancer patients are hormone receptor‐positive, antiestrogen drug conjugates hold promise as effective therapeutics.^32^ Although some tamoxifen drug conjugates display higher potency compared to tamoxifen alone, there remains the risk of tamoxifen drug resistance. Therefore, testing these conjugates in tamoxifen‐resistant breast cancer lines is critical. A recently reported series of tamoxifen‐melatonin conjugates (US Patent No. 08785501) hold promise against ER, HER2, triple‐negative, and tamoxifen‐resistant breast cancer cells (to be published). Clinical studies need to be performed to determine if drug conjugates demonstrate greater convenience for patients who are prescribed multiple medications resulting in better patient management by the physician. With safety and efficacy always being major concerns in anticancer treatments, clinical studies need to determine if drug‐drug conjugates offer an advantage over cancer therapies utilizing 2 unlinked drugs by improving pharmacokinetic and pharmacodynamic drug profiles; all of these may mitigate the toxic bystander effect and spare healthy neighboring cells. These collective features need to be further studied, both preclinically and clinically to determine if they result in superior safety profiles and patient outcomes.

All drug conjugate classes hold promise, whether they are linked to antibodies, radionuclides, or nanoparticles. Indeed, 2 clinical trials are currently assessing the efficacy of CDX‐011, a glembatumumab vedotin antibody‐drug conjugate against triple‐negative breast cancer (NCT01997333), and SYD985, a trastuzumab‐duocarmazine antibody‐drug conjugate against HER2 breast cancer (NCT03262935). Thus, drug conjugates are emerging as a novel and effective treatment approach for breast cancer.

## AUTHORSHIP CONTRIBUTIONS

Conception of review (MH; PWE); Writing of manuscript (MH; DPZ; PWE); Reviewed/Edited/Revised (PWE; RKL; RES).

## DISCLOSURE

US Patent 08785501.
